# Auditory feedback of one’s own voice is used for high-level semantic monitoring: the “self-comprehension” hypothesis

**DOI:** 10.3389/fnhum.2014.00166

**Published:** 2014-03-28

**Authors:** Andreas Lind, Lars Hall, Björn Breidegard, Christian Balkenius, Petter Johansson

**Affiliations:** ^1^Department of Philosophy, Lund University Cognitive Science, Lund UniversityLund, Sweden; ^2^Certec - Division of Rehabilitation Engineering Research, Department of Design Sciences, Lund UniversityLund, Sweden; ^3^Swedish Collegium for Advanced Study, Linneanum, Uppsala UniversityUppsala, Sweden

**Keywords:** speech production, self-monitoring, feedback manipulation, auditory feedback, real-time speech exchange, self-comprehension, agency

## Abstract

What would it be like if we said one thing, and heard ourselves saying something else? Would we notice something was wrong? Or would we believe we said the thing we heard? Is feedback of our own speech only used to detect errors, or does it also help to specify the meaning of what we say? *Comparator* models of self-monitoring favor the first alternative, and hold that our sense of agency is given by the comparison between intentions and outcomes, while *inferential* models argue that agency is a more fluent construct, dependent on contextual inferences about the most likely cause of an action. In this paper, we present a theory about the use of feedback during speech. Specifically, we discuss inferential models of speech production that question the standard comparator assumption that the meaning of our utterances is fully specified before articulation. We then argue that auditory feedback provides speakers with a channel for high-level, semantic “self-comprehension”. In support of this we discuss results using a method we recently developed called *Real-time Speech Exchange* (RSE). In our first study using RSE (Lind et al., in press) participants were fitted with headsets and performed a computerized Stroop task. We surreptitiously recorded words they said, and later in the test we played them back at the exact same time that the participants uttered something else, while blocking the actual feedback of their voice. Thus, participants said one thing, but heard themselves saying something else. The results showed that when timing conditions were ideal, more than two thirds of the manipulations went undetected. Crucially, in a large proportion of the non-detected manipulated trials, the inserted words were experienced as self-produced by the participants. This indicates that our sense of agency for speech has a strong inferential component, and that auditory feedback of our own voice acts as a pathway for semantic monitoring. We believe RSE holds great promise as a tool for investigating the role of auditory feedback during speech, and we suggest a number of future studies to serve this purpose.

## Introduction

In the study of action and agency there has been a vigorous debate between *comparator* and *inferential* models (Daprati et al., 2003; Haggard and Clark, 2003; Synofzik et al., 2008; Kühn et al., 2013). According to the comparator perspective, comparisons between intentions and outcomes (and comparisons between intentions and the predictive simulations of outcomes) is what anchors our fundamental sense of self as agents, and allow us to source monitor between actions that are generated by ourselves, or done by others (Gallagher, 2000; Blakemore et al., 2002; David, 2012; Kühn et al., 2013). Furthermore, a comparator architecture is what is supposed to underlie error correction by giving us the ability to discriminate deliberate from accidental outcomes (Frith, 2014), and what we have done from what we plan to do (Sugimori et al., 2013). On the other hand, inferential theories have noted that there are a variety of abnormalities of authorship processing, occurring in both natural and experimental conditions (such as alien hand syndrome, schizophrenia, hypnosis, etc.), that suggest our sense of agency is a much more fluent and fragile construct that is dependent on inferences about which agent was the most probable cause of the action, and what purpose or meaning the action had (Wegner and Wheatley, 1999; Moore et al., 2009). As Wegner and Sparrow (2004) puts it:

These examples suggest that authorship knowledge is not a “given” when people produce apparently voluntary actions, and instead that resolving the question of authorship for any action may require considerable information and inference. Authorship processing is a form of causal inference in which events are attributed to entities that are perceived to cause them (p. 1202).

It is natural to assume that this interesting clash of perspectives would be mirrored closely in the study of speech production and verbal self-monitoring, but surprisingly there has been very little experimentation and discussion regarding the potential role of auditory feedback in determining what we say and mean (Dennett, 1991). This unfortunate lack of investigation stems from the fact that all dominant theories of speech production are part of the comparator family of models, and share the assumption that speech starts with a clear preverbal conception of what to say, which is then translated into an utterance through successive levels of linguistic and articulatory encoding. However, in a recent study (Lind et al., in press), we found evidence to suggest that our sense of agency for speech has a strong inferential component, and that auditory feedback of our own voice acts as a pathway for semantic monitoring, potentially overriding other feedback loops. In this paper we present an inferential theory about the use of auditory feedback in which we believe the results of Lind et al. (in press) become comprehensible, rather than unintuitive, as present theories of speech production and self-monitoring would suggest. Specifically, we propose a model of speech production that questions the standard assumption that speech is always guided by speech plans which are so detailed that the meaning of speakers’ utterances are fully specified before articulation. We then argue that auditory feedback is a prime channel for monitoring high-level semantics, allowing speakers to perform a form of continuous “self-comprehension” of their speech. Finally, we present suggestions for future work using the method we have developed.

## The standard translation model of speech

If one considers speech to be principally a top-down affair where a speech plan is first specified in detail and then translated through temporally and locally separated stages into an actual utterance, then it is very close at hand to assume that speech feedback play only a supervisory role. The speaker knows what is to be said and via feedback she makes sure that the machinery does not “glitch” at any of the translation stages.

Underlying this view of self-monitoring as comparison of output with plan we find what has been called the *translation* view of speech production (by e.g., Fowler, 1980; Fowler et al., 1980; Linell, 1982). This is the standard type of speech production model. The basic idea is that speech is governed by a plan constituted by high level abstract invariant elements which then needs to be translated into physical articulation (e.g., Fodor et al., 1974; Shattuck-Hufnagel, 1979; Dell, 1986; Levelt, 1989; Dell et al., 1993; Levelt et al., 1999). Consequently, the task of psycholinguistic models of speech production has for a long time been seen as the task of accounting for the successive steps in this translation process. The basic framework in modern theories can be traced back to Lashley (1951), whose idea of *planned behavior* represented an alternative to the prevailing behavioristic framework. His general idea was that there are underlying plans for action sequences and he appealed to typing and speech errors as evidence of the existence of such plans. Errors are thought to reveal how a plan must exist, which is then for some reason not correctly carried out. For example, in an anticipation error, a segment which is to be spoken later in a sequence mistakenly substitutes for a segment earlier in the sequence, such as in “John dropped his cuff of coffee” (taken from Fromkin, 1971). Indeed, the analysis of speech errors was for a long time the main empirical tool for constructing models of the translation processes believed to go on in-between the message to be transmitted, and the actual (motoric) articulation of the utterance (e.g., Fry, 1969; Fromkin, 1971; Garrett, 1975, 1976, 1980). The types of errors that speakers make, including the types of segments which are believed to be open to exchange, deletion, or addition have been used to construct arguments about the specific workings of the translation processes. For example, an error at the level of the phoneme motivates the postulation of a specific level of processing that deals with the allocation of phonemes.

This enthusiasm over error analysis has contributed to postponing the study of higher levels of speech planning. While translation models differ in details, an important commonality is that there is virtually no speculation about how the content of a message to be conveyed is formulated. Rather, it is just assumed that it *is* specified, and that it can provide the necessary impetus for the rest of the production apparatus. Similarly, the specifics of articulation are also seen as peripheral to the modeling. Levelt et al. (1999) admit this themselves: “Our theory of lexical access is not well developed for this initial stage of conceptual preparation” (p. 8). Avoiding this question of, as Dell (1986) puts it, why a speaker says what is said, and focusing instead on how it is said, can be seen as a pragmatic strategy, allowing the researcher to focus on aspects of production where it is easier to acquire empirical evidence. However, as we see it, the very structure of this type of model has made it too convenient for researchers to simply disregard the supposed abstract high level by assuming that too little is known about it, and that we will do better to focus on the more tangible translation process. So while Levelt (1989) admits that the conceptualizer is a simplification that needs to be further explored, he also makes clear that “the mother of each speech act is a communicative intention” (1989, p. 108), and simply asserts that “where intentions come from is not a concern of this book” (ibid., p. 59). Similarly, in his comprehensive summary of speech production models, Postma (2000) simply takes as his starting point that specific preverbal messages needs to be formulated: “Speaking starts with conceptualization (planning an utterance’s meaning and purpose). The conceptualizer delivers a propositional, preverbal message to the formulator. The formulator translates the preverbal message into a linguistic structure” (p. 99). The result of these assumptions has been that the division between abstract and physical aspects of speech has been solidified, and the delegation of the process of conceptualization to a dedicated black box “conceptualizer” has not really been questioned.

This general structure is explicitly or implicitly accepted by most models of speech production. When Hickok (2012, 2014) calls for the integration of traditional psycholinguistics and more motor oriented theories of speech, he argues that both fields are dominated by the same family of models. In particular, a commitment to a comparator architecture is evident in the currently extremely popular notion of forward models in speech (e.g., see Heinks-Maldonado et al., 2005, 2006; Guenther et al., 2006; Christoffels et al., 2007; Behroozmand et al., 2011; Chang et al., 2013; Chen et al., 2013; Greenlee et al., 2013; Houde et al., 2013; Nelson et al., 2013; Pickering and Garrod, 2013). As Chen et al. (2013) puts it:

It has been suggested that the error signal that results from a mismatch between the forward model prediction and the actual sensory feedback enables the audio-vocal system to distinguish self-produced speech from externally-generated sounds, to correct for vocal errors during ongoing speech production, and to optimize the internal model for future productions (p. 2).

## An alternative view on speech production

Despite its widespread dominance, critiques of the translational model can be found from many different perspectives (e.g., Harris, 1981; Kelso and Tuller, 1985; Sperber and Wilson, 1986; Elman, 1995; Goldstein, 2004). There are empirical findings that are difficult to reconcile with a translational framework. For example, spectrograms have revealed how there are no invariant realizations of the supposed high-level segments believed to go into speech planning (see Casserly and Pisoni, 2010, for a historical overview). Due to this invariance at the physical level, the translation account has been accused of imposing a mind-body dualism (see e.g., Hammarberg, 1976; Fowler, 1980). It has also been shown that speech errors often involve activation of muscles aiming towards both the (supposedly) intended and the unintended speech sounds (e.g., Mowrey and MacKay, 1990). Both these findings have been taken to indicate that speech errors should not be seen as the displacement of high-level, abstract segments, and that planning and articulation are not as separate and hierarchically ordered as is assumed in translation theories.

The view of the individual speaker which we find in the translation model constitutes the speaker part of the so called “speech chain” (see Denes and Pinson, 1963). The speaker has a message, which she translates into sound waves that hit the ear of the listener, who then reconstructs the message, formulates a response, translates it into sound waves, etc. This also corresponds closely to the standard folk-psychological notion of language use. Reddy (1979) points out that the way we speak about language in everyday situations reveals that the concept of “thought transfer” is deeply embedded in our folk-psychological view of language. A number of interrelated claims in this approach are that the speaker is (i) considered in relative isolation; (ii) that the meaning of the utterance is to be found solely in the intentions of the speaker; (iii) that cognition comes before communication; and (iv) that contextual factors are seen as largely external to language (see Linell, 2009).

An alternative way of approaching language is from an explicitly “dialogical” stance. Here, spoken language is seen as inherently interactional and context-bound, and meaning as actively co-created with other speakers (e.g., Linell, 1998). For example, Vološinov (1986) has argued that the meanings of our utterances are inseparable from the contexts in which they are uttered. Cognition and communication are not seen as separate, temporally successive stages of interaction, but rather as two aspects of the same thing (Linell, 2009). Accordingly, meaning in spoken language is seen as embedded in the environment, in the conversational context, in the interaction with other speakers etc. For the individual speaker, this means that the meaning of her words is not fully determined prior to taking linguistic form in actual speech acts. Linell (1979) states that:

In most cases of normal spontaneous conversation we start speaking without actually knowing precisely what we are going to say. […] This suggests a theory according to which the communicative intentions are partly imprecise, vague and preconscious from the start, and in which they become gradually more structured, enriched, precise and conscious through the verbalization process itself (p. 10).

Just as Wegner and Sparrow (2004) argue that authorship knowledge is not something “given” on the inferential account, the dialogical model argues that the full meaning of the utterance is not given to the speaker prior to the actual utterance. Instead, it places more importance on speech feedback to allow the speaker to draw inferences about what, precisely, she is communicating. Most likely, this includes cues and feedback from the social situation: from the context of speech, from the environment and from her interlocutors’ responses.

### Cognitive modeling compatible with the alternative view

There have been some suggestions about how the cognitive processes that generate meaningful utterances can be modeled, which we believe are capable of accommodating the view of the speaker as flexible and sensitive to contextual factors in the speech situation. For example, Dennett (1987, 1991) criticizes the tendency to posit central executives in psychological models. In discussing Levelt’s model, he questions the reliance in speech production research on an all-knowing and powerful homunculus-like conceptualizer to deliver completed meanings to the production system:

The problem with [the serial model] is that the Conceptualizer seems ominously powerful, a homunculus with too much knowledge and responsibility. This excess of power is manifested in the awkward problem of how to couch its output, the preverbal message. If it already specifies a speech act […] most of the hard work of composition has happened before our model kicks in (Dennett, 1991, p. 238).

To provide an account of speech production that actually targets the problem of conceptualization, Dennett (1991) instead proposes a distributed, or pandemonium, model. Rather than postulating a conceptualizer, his model involves a competition for control over the production processes between different specialized circuits. The content of speech is assumed to be determined in an opportunistic fashion as power is shifted around in a “quasi-evolutionary process”, at each point producing several different variations on how an utterance could be formed. Rather than postulating fully specified intentions, Dennett uses the concept of a “mind-set” which functions as a constraining mechanism on these processes. Proposals are, usually non-consciously, but sometimes consciously, weighed against each other in a “collaboration […] of various subsystems none of which is capable on its own of performing—or ordering—a speech act” (ibid., p. 239). This allows the speaker to eventually zero in on a specific proposal for how the utterance should be performed. These competitive processes take place on a scale of milliseconds, meaning that the content of the speech act is not fully specified until it is actually spoken, and that we often learn the specific content of our speech act as we speak.

A similar type of model is Baars’ (1980) *competing plans hypothesis*, which assumes that multiple plans are often developed during speech production. He holds that it represents a necessary feature of any control system, and he claims that it can ultimately account for the enormous flexibility of the speech production system. In outline, he proposes that executive systems focus their limited capacity on approximate orders that are “not *fully* elaborated” (rather, they are somewhere within a “ball-park”, see p. 46) and multiple, competing, plans may be developed, amounting to much the same thing, but with slight variations, e.g., in shades of meaning. The full, detailed, elaboration and realization of these approximate, vague, and possibly ambiguous plans are then carried out by specialized, “intelligent, semi-autonomous subsystems” that are themselves beyond direct executive control (p. 49).

## Real-time speech exchange

While we believe the alternative accounts developed by the likes of Dennett, Linell and Baars serves as important counterpoints to the dominant translation/comparator model of speech, the field of psycholinguistics still suffers greatly from a lack of direct empirical investigation of the conceptualization process. Thus, in an attempt to approach the issues of speech production and self-monitoring from a semantic perspective, we have developed a new research methodology we call *Real-time Speech Exchange* (RSE; Breidegard et al., in preparation; Lind et al., in press) for technical details), which allows us to create situations where participants say one thing, but they receive real-time auditory feedback of their own voice suggesting that they are saying something else.

According to the dominant comparator perspective on speech, the marked discrepancy in meaning between what participants actually say and what they hear themselves saying should be detected as originating from an external source. As Weiss et al. (2013) states it: “…the higher the discrepancy, the more an action is experienced as caused not by oneself, but by another cause, such as another agent” (p. 2). Similarly, as stated by Sugimori et al. (2013):

According to the forward model… speech is regarded as emanating from the self only when the actual feedback matches the prediction. That is, the efferent copy issued from the intense speech command and appropriate feedback are needed to obtain a sense of agency over speech (p. 361).

In contrast to previous perturbation studies in the speech literature, the manipulations in our recent study (Lind et al., in press) create both distinct semantic mismatches and carry direct contextual consequences, such that if the participants believe themselves to have uttered the inserted word, they will also believe themselves to have made a mistake during the experimental test. On the other hand, according to the inferential model, auditory feedback is actively used for self-comprehension. Thus, on this account it would be expected that the participants accept the inserted statements to be self-produced, and believe they have committed an error on the given trial.

**Figure 1:**
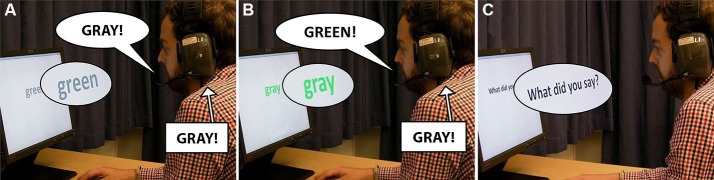
**Example of a recording and manipulation (in this case from “green” to “gray”) during the Stroop test**. **(A)** At a non-manipulated trial, a word (e.g. “gray”) is recorded. The participant receives auditory feedback of the word he is uttering. **(B)** Later in the test, the participant’s feedback is manipulated by inserting the recording of “gray” as the participant utters the word “green”. **(C)** The participant is asked “what did you say?” directly following the manipulation.

In Lind et al. (in press), participants performed a computerized Stroop test (Stroop, 1935). In the Stroop test you are shown color words printed in a specific color (such as **BLUE**), and the task is to always name the printed color, while ignoring the spelled word. In the experiment we were seated in a hidden control room, covertly controlling the PC-based voice exchange program using a computer-game gamepad. For each manipulation, we first recorded specific color words that participants uttered. The program automatically cropped these recordings along the time axis, so that word onset perfectly matched the onset of the recording (each cropping was also checked and if necessary corrected manually by the experimenter using a spectrogram-presentation). Later in the test, we enabled a trigger mechanism which automatically inserted the appropriate pre-recorded color-word into the participant’s feedback, and simultaneously, the program blocked the feedback of what the participant was actually saying. To achieve this, participants wore headsets constructed from highly sound isolating ear muffs fitted out with a microphone and loudspeaker transducers, and we set the sound levels at 8–10 dB above normal speaking level (as measured from the ear of the speaker), effectively masking any air-conducted sound of the speaker’s voice which may leak through the headphones. For example, during a manipulated trial, the participants might have seen the word “gray” in green color. They correctly said “green” (“grön” in Swedish), but heard themselves say the phonologically similar, but semantically distinct, word “gray” (“grå” in Swedish) (see Figure 1). As the latency in the voice exchange software was as low as 8 ms, this allowed us to create voice exchanges with usually very high timing accuracy. The participants were also instructed to use the same tone of voice during the whole experiment, which increased the probability that the spoken and the inserted words were similar in pronunciation. Inserted words were recorded in as close proximity to the manipulations as possible. This means that whatever differences there would be between a participant’s pronunciations at different stages of the test, these differences would be present both in the spoken and the inserted word.

Seventy-eight Swedish-speaking participants performed the 250 trial randomized Stroop test, which took approximately 10 min to complete. There were four manipulations during the test, 2 from “gray” to “green” and 2 from “green” to “gray”. In order to investigate participants’ experience of each manipulation, we stopped the test directly after a manipulated trial and the question “What did you say?” was presented on the screen. When the participants had answered, the test resumed. Spread out in between manipulated trials there were also four stops where the test stopped and the question was presented, but without there having been a manipulation beforehand. This allowed us to make sure that participants had no trouble answering the questions in general.

There were two methodological reasons why we found the Stroop task particularly fitting for this initial experiment. First, RSE requires that we know in advance what the participants are about to say, otherwise it would be impossible to precision fit a replacement utterance. Our Stroop variant was run at an unhurried pace, and there was a baseline of only around 2% errors in the test. Thus, the responses of the participants at each trial are easy to predict for the experimenter who has to perform the recording and playback procedure. The second main reason we chose to utilize the Stroop task was that it represents a technically manageable scenario where two alternative answers (the print color or the color word) are at least possible as candidate utterances. While the effect of a manipulation was to present a highly unlikely error to the participants, it was still contextually possible that they might have said the inserted word.

We performed structured post-test interviews to determine if the participants had detected the manipulations, how many they had detected, and how the manipulations were experienced. As a general rule, we took the participants’ reports at face value when determining number of trials detected, i.e., if a participant says he or she detected three manipulated trials we assume that this is a correct estimate. We then used various cues to determine which of the manipulated trials were detected, such as seriously mistimed trials detected by the participant, or identifiable comments about their own behavior (e.g., “the first time I detected a manipulation I answered the word I heard in my earphones, but the following two times I gave the correct answer instead”). We classified the detections into three levels of certainty ranging from total certainty and a full understanding of the manipulation procedure, to an uncertainty as to if anything out of the ordinary had even occurred. If participants detected an exchange, they were alerted to the occurrence of external manipulation and the purpose of the experiment. The test then changed to an explicit mismatch detection task, and given the low baseline error rate of the Stroop test, it was easy to self-monitor based on the objective criteria of correctness in the task. To avoid any such confounds, we removed all trials following a first detection (resulting in the removal of 129 trials; see Lind et al., in press, for more details regarding the procedure). While we managed to make a majority of voice exchanges with high timing accuracy, some manipulations had sub-optimal timing in that the inserted feedback either preceded or followed what the participants actually were saying. We used this to investigate the impact of timing accuracy by comparing manipulations within a timing window of 5–20 ms (including the 8 ms delay) with all other manipulations (delayed auditory feedback starts affecting speech at around 30 ms, see e.g., Lee, 1950; Takaso et al., 2010).

The results showed that under ideal timing conditions, we found a level of detection as low as 32%, with no more than 4% being certain of their detections (there were no differences between the two types of exchanges, that is from “green” to “gray” or from “gray” to “green”). Thus, when near simultaneous timing-conditions are met, very few participants had more than a vague hunch that what they heard themselves say was not what they actually said. The importance of the timing window we established was confirmed by the data. When the timing was off, 55.5% of the manipulations were detected, significantly more than when the timing was good. Furthermore, the proportion of certain detections was considerably higher when the timing was off (24%, compared to 4% when the timing was good), which further emphasizes the importance of timing. Describing the overall result in participant percentages, and combining timed and mistimed trials, 61.5% of the participants failed to detect at least one of the manipulated trials.

So, when the conditions we set up for our experiment were fulfilled, many of the word exchanges were not detected. But how did the participants react to the question “*what did you say?*” When the question was posed after a non-manipulated trial, participants answered the question according to what they had said 99.4% of the time, so the baseline was to make virtually no mistakes here. Responses to the non-detected manipulated feedback, however, indicated that participants often accepted the inserted feedback as if it had been self-produced. In 38.5% of the non-detected trials they simply answered what they had heard. For example, if they had said “green” and heard themselves say “gray”, they answered “gray” when asked what they had said. As it was easy to self-monitor based on the objective criteria of correctness in the task (i.e., participants could remember the correct answer from the visual representation on the screen), a number of participants also accepted the inserted words as self-produced, but “corrected” themselves in various ways. In 16.5% of the non-detected trials they did so either spontaneously before the question even appeared on the screen (for example they said “green”, heard “gray”, and immediately they said “no, green”), or by correcting themselves when answering the question (for example, they said “green”, heard “gray”, and then when the question popped up they answered it “I mean “green””). In another 29.7% of the non-detected trials participants answered the question according to what they actually said, but during the post-test interview revealed that they believed themselves to have made a mistake on those trials. That is, they accepted the inserted word as self-produced, but they answered the question according to what they thought was the correct answer in the test. Summing up these categories we found that in a full 85% the non-detected manipulated trials, the participants actually believed they had said the word that we had played back to them. In the remaining 15% of non-detected manipulated trials, participants answered the question according to what they had actually said, but we were unable to determine how participants actually experienced the feedback manipulations.

In our previous research on the phenomenon of choice blindness (CB) we have contributed evidence to the effect that knowing one’s own attitudes is an inferential process, and that we cannot simply introspect why we choose and act the way we do. CB is a choice paradigm originally inspired by techniques from the domain of close-up card-magic, which permits us to surreptitiously manipulate the relationship between choice and outcome that our participants experience, which has allowed us to demonstrate that participants often fail to notice mismatches between what they choose and what they actually get (hence, being blind to the outcome of their choice) (e.g., Johansson et al., 2005, 2006, 2013; Hall et al., 2012, 2013). The results of Lind et al. (in press) indicate that speech intentions similarly have a strong inferential component.

On the other hand, currently dominant theories of speech production and self-monitoring would regard these results as surprising and counterintuitive. We know from previous studies using feedback manipulation techniques that speakers will sometimes compensate for perturbations of e.g., voice fundamental frequency (*F*_0_) or formant frequencies in their auditory feedback by shifting their production in the opposite direction (see Section Different Feedback Channels Monitor Different Aspects of Speech below for more on this), indicating that they accept the feedback as being self-produced. But it has also been found that when manipulations exceed a certain magnitude, compensation decreases (MacDonald et al., 2010), as does neural responsiveness to the perturbations (Behroozmand et al., 2009). This suggests that speakers now process the perturbations as coming from an external source instead, similar to how visual/manual illusions such as the “rubber hand” illusion collapses when the magnitudes of specific aspects of the manipulations become too large (see MacDonald et al., 2010, p. 1066, for a discussion). In line with this, the manipulations in the current experiment have distinct semantic and contextual consequences in that, if the speaker believes herself to have uttered the inserted word, then she will also believe herself to have made a mistake during the trial. Thus, from a comparator perspective the semantic level mismatch between intention and outcome generated by the manipulations should signal a lack of agency, and guarantee that the exchanged words would be detected as coming from an external source (e.g., see Fourneret and Jeannerod, 1998, for a similar discussion in the manual domain).

## Feedback during speech

As discussed above, we believe the result of Lind et al. (in press) fits nicely with theorists who emphasize the social nature of language and a more actively interpretative use of feedback, and hold that the meaning of one’s own utterances are not necessarily fully clear before they have been said.

But how do our results relate to previous studies on feedback processing in speech? In this section we will look at research showing that feedback is crucial for controlling several aspects and levels of our speech. We will argue that different channels of feedback are used for monitoring different aspects of our own speech. In the next section we will then propose that a main function of auditory feedback is that it is used in a form of online “*self-comprehension*” which allows speakers to internalize the context-bound consequences (i.e., meanings) of their utterances.

### Different feedback channels monitor different aspects of speech

Researchers have long tried to specify what types of feedback help the speaker in controlling her speech output. Ladefoged (1967) suggested that different channels of feedback are responsible for monitoring different aspects of speech:

The speaker has three kinds of feedback about the sounds he is producing: auditory feedback by means of both bone and air conduction; tactile feedback about the contacts between the lips, tongue, velum and other parts of the vocal tract; and kinesthetic feedback about the stretch of the muscles and the movements of the joints. Many aspects of speech may be controlled as a result of information available through more than one of these feedback channels. But it seems that certain aspects are monitored typically via one channel rather than another (pp. 162–163).

Empirical work on the uses of proprioceptive and auditory feedback suggests this picture is largely correct. There is evidence to suggest that somatosensory information provides a robust and independent frame of reference for articulation. For example, we know from post-lingually deafened individuals that even if some aspects deteriorate, speech can remain intelligible for a long time after loss of hearing (e.g., Cowie et al., 1982; Waldstein, 1990; Lane and Webster, 1991). Furthermore, studies have shown how speakers compensate for jaw position perturbations even when these have no acoustic consequences, indicating that the somatosensory feedback can aid control of speech movements without the help of auditory feedback (Tremblay et al., 2003; Nasir and Ostry, 2006; but see also Feng et al., 2011).

But this does not mean that auditory feedback is not used extensively when it is available. A classic example of the importance of the auditory loop is the “Lombard effect” (Lane and Tranel, 1971; Patel and Schell, 2008), which deals with the largely automatic (Pick et al., 1989) adjustments speakers make as a response to noise in the environment, and which results in more intelligible speech under such circumstances. More recently, inventively designed experimental studies using real-time perturbations of auditory feedback have shown how a variety of aspects of speech production are sensitively tuned to the feedback that speakers receive of their own voice. For example, Houde and Jordan (1998, 2002) showed how speakers compensate for formant shifts (*F*_1_ and *F*_2_) that were gradually induced in the auditory feedback of their whispered speech. Villacorta et al. (2007) showed similar results for *F*_1_ during voiced speech (see also Purcell and Munhall, 2006a,b). Compensation has also been shown for suddenly (Burnett et al., 1997, 1998; Purcell and Munhall, 2006a) and gradually (Jones and Munhall, 2000, 2005) induced changes in *F*_0_, and for suddenly induced perturbations in *F*_2_ in a multisyllabic utterance (Cai et al., 2011). These types of compensation appear to be automatic and reflexive (see Munhall et al., 2009; Isius et al., 2013), and very difficult to suppress even when participants are instructed to do so (Keough et al., 2013). While it is mainly vowels that have been the focus of perturbation studies, there are indications that stop consonants and [s] fricatives are also monitored using auditory feedback (Waldstein, 1990; Shiller et al., 2009 respectively; though see Casserly, 2011).

Ladefoged (1967) further speculated that when one type of feedback is absent for some reason, other channels of feedback may take over those specific functions. There are indications that multiple monitoring channels can take part in monitoring specific aspects of speech and that if one channel is absent then other channels may become more dominant. For example, there is evidence to suggest that control over *F*_0_ is maintained using both proprioceptive and auditory feedback (Larson et al., 2008). Importantly, Larson et al. (2008) showed how pitch-shifted feedback elicited greater compensatory reactions when feedback from the vocal folds was removed with anesthesia. This suggests that when incongruent proprioceptive feedback was not there to counter the manipulation of the auditory feedback, the effect was greater since the speakers had to rely solely on the auditory feedback.

Relating this to the result of Lind et al. (in press), during the manipulated trials the proprioceptive (and bone-conducted) feedback of participant’s utterances was inconsistent with the auditory feedback. The fact that participants nevertheless often believed themselves to have uttered the word that was inserted into their feedback indicates that when it comes to the meaning of utterances, auditory feedback can override proprioceptive feedback (Feng et al., 2011, similarly found evidence that auditory feedback plays a primary role during speech monitoring, relative to proprioceptive feedback). To our knowledge, no previous studies investigated the role of feedback on the actual *meaning* of spoken communication. We believe our result adds an important aspect to the view of differential speech monitoring, namely that auditory feedback could be seen as the primary channel for high-level semantic monitoring.

This interpretation can be contrasted with a central feature of the influential Levelt comparator model. One aspect of this model that has been adopted by many other researchers is the postulation of an internal monitoring channel (e.g., Levelt, 1983, 1989; Levelt et al., 1999; Özdemir et al., 2007). According to Levelt, this internal channel uses the comprehension system in much the same way as the external channel does, but it instead monitors an “articulatory buffer” which holds phonological (e.g., Levelt, 1989), or later phonetic (Levelt and Wheeldon, 1994; Wheeldon and Levelt, 1995) plans. This buffering mechanism was postulated because the encoding of an utterance is believed to be completed quicker than it can be articulated. However, we believe the reliance on auditory feedback shown in our experiment suggests that either this postulated internal channel is unavailable during overt speech (as has been suggested by Vigliocco and Hartsuiker, 2002; Huettig and Hartsuiker, 2010; Nozari et al., 2011), or that the external channel has so much primacy that it can override the internal one.

### Speaking-induced suppression

In neurocognitive investigations of feedback, it has been shown how responses in the auditory cortex are reduced during overt speech when speakers receive unaltered feedback of their own voice, compared to when they receive pitch-shifted feedback or when they listen to recordings of the same utterances (e.g., Curio et al., 2000; Houde et al., 2002; Heinks-Maldonado et al., 2005, 2006). This effect of speaking-induced suppression (SIS) has been taken as evidence that the auditory cortex anticipates the effects of utterances, suggesting a forward model mechanism for speech production. As we discussed in the introduction, this is supposed to give speakers a sense of agency for their speech, and separate self-produced sounds from sounds coming in externally.

The presence of SIS provides clear evidence that the speech system has some privileged knowledge about what is about to be uttered in comparison to listeners. However, it has not been shown that this knowledge is fully represented and specified at the semantic level. Our findings with RSE (Lind et al., in press) suggests caution is warranted in going from SIS and forward models of motor loops to a similar architecture at the level that concerns what we intend and decide to say (e.g., Hickok, 2012, 2014; Pickering and Garrod, 2013). We suggested previously that utterances are given full meaning first when they are actually uttered and interpreted within their context of use. While the details of articulatory motor programs may be specific enough to elicit alarms when there is a mismatch with actual performance, processing the meaning of an utterance might require a context which is to a considerable degree external to the speaker’s nervous system (see also e.g., Howes et al., 2013, who express similar skepticism regarding the specificity of forward modeling during speech).

In this context it is interesting to mention the findings of Ventura et al. (2009). They investigated the impact of utterance complexity and speed of speech upon SIS, using three different speech conditions: /a/, /a-a-a/, and /a—a-a—a/. They found that the more complex and rapidly spoken the utterance was, the less pronounced the SIS was (that is, the difference between speaking and listening to a recording of the same speech was greater the simpler the utterance was). This indicates that as speakers move from single-vowel utterances, which are what mostly has been used in the SIS literature, to more complex vowel sequences, then SIS is reduced. Ventura et al. (2009) interpret this as follows: “The greatest SIS was observed […] with the simple utterance presumably because the internal representation, or mental model, for that utterance was largely static and therefore easy to produce and match” (p. 5). From this, it seems to follow that that the more rapid and complex the utterance is, the less specified the efference copy is, and the more speakers might have to rely on inferential processes of self-comprehension. We believe that the inclusion of the complex contextual semantic aspects of even the most basic utterances (as when naming colors in a Stroop task) should further add to the need for feedback to determine what one has said. In normal speech, utterances are a great deal more rapid and complex than the rhythmic vowel-sequences used by Ventura et al. (2009).

## Self-comprehension

In Lind et al. (in press) we showed how speakers often failed to detect when we exchanged the auditory feedback of their own speech to phonologically similar, but semantically distinct, words. Furthermore, participants often accepted these inserted words as if it had been they themselves who had uttered them. In this paper we have discussed models of speech production and empirical work on the use of feedback during speech, with an eye towards making our surprising results understandable.

The main hypothesis presented in this paper is that auditory feedback of our own speech is not just used to make sure we say precisely what we intended to say. Rather, we propose that our utterances often are semantically underspecified, and that we actively use feedback to help specify for ourselves the full meaning of what we are saying. In effect, we propose that auditory feedback provides us with a channel for high-level, semantic “self-comprehension”. As Linell (1998) puts it: “Speakers talk not only in order to be understood by their interlocutors, but also in order to understand what they themselves say and think. The speaker is also a recipient of his own utterance” (p. 94).

However, in all feedback manipulation studies, it is necessary to assure a high degree of control over the experimental situation. This usually includes instructing the participants what to say and when to say it (e.g., by displaying target words on a computer screen). It is extremely difficult to create experimental situations that allow researchers to manipulate specific aspects of speakers’ feedback in spontaneous speech (but see below for suggestions on future RSE studies). Therefore, we do not know to what extent the results of Lind et al. (in press) transfer to everyday language use (see e.g., Borden, 1979, for a discussion about how feedback manipulation experiments might, or might not, reveal functions of feedback outside the laboratory). We chose to use the Stroop task for the initial study partly because of the predictability it provides, but also because a Stroop trial has two potential answers: either you correctly name the color, or you mistakenly name the spelled word. This is necessary since if there had been only one possible answer (if, for example, participants were to read color-words written in black and we had exchanged e.g., “green” to “gray” in their auditory feedback), then the inserted word would have made absolutely no sense contextually, and the self-comprehension model would predict that the participants would infer that the utterances were not self-produced. Yet, while the inserted words constitute potential answers in the Stroop test, participants easily avoided errors in the test (as mentioned, the error rate was below 2%). This means that the “errors” we induced on manipulated trials constituted very unusual responses for the participants. But the inserted feedback was accepted as self-produced in spite of their records of correct answers. If participants can accept inserted feedback as self-produced even when this indicates that they have made a much more improbable response, this suggests that we also can expect manipulations to be accepted as self-produced in natural speech, where there is a much broader range of possibly appropriate answers. To see this, compare the favorable conditions for self-monitoring in a situation where we are explicitly instructed what to do, and can easily remember the correct response from the screen, with the contextual ambiguity and uncertainty of expressing our opinions on the conflict in Syria during a fluid dinner conversation (e.g., see Hall et al., 2012, 2013, for examples of how remarkably flexible our expression of moral and political attitudes can be).

Importantly, the self-comprehension model does not deny that error correction exists. The difference between our view and the standard view lies in how the decision that a word was erroneously uttered was made. According to the standard view, the decision is made on internal criteria, that is, the speaker’s original intention provides the benchmark for correctness (see e.g., Postma, 2000). We instead suggest that self-comprehension, which relies heavily on external criteria from the whole conversational context, provides the necessary information for the speaker to judge if her speech was a mistake or not. This high level comprehension based correction is supplemented by lower-level articulatory error correction, such as for prevoicing and timing alterations (e.g., Cai et al., 2011).

“Self-comprehension” is implied in previous monitoring theories (such as Levelt, 1989), where the comprehension system is used as we listen to ourselves for monitoring purposes. Supposedly, this listening is using the comprehension systems in much the same way as when we listen to others speaking. Our hypothesis is that we use auditory feedback of our own voice in order to fully understand what it is we are saying, while in comparator models like Levelt’s, the full meaning of the utterance is already fixed as the articulation takes place, and self-comprehension is seen as just a prerequisite for self-monitoring. However, this does not mean that we believe that self-comprehension is every bit like the comprehension of others. It would be ridiculous to deny that people have the capacity to plan and mentally simulate outcomes (linguistic or otherwise) before executing an action. For example, Pickering and Garrod (2013) argue that speakers routinely make predictions about what they will say during speech, and that speakers base these predictions on an efference copy of the speech command. In line with this, studies of SIS and automatic compensation in feedback perturbation clearly indicate that (some parts of) the effects of the utterances are expected by (some parts of) the speech production system. But efference copies are not prohibited by the self-comprehension model, only the notion that they serve as the exclusive standard for the final meaning of our utterances. Unfortunately, there has been very little discussion in the speech perturbation literature about levels of adaptation and consciousness (in contrast to the manual domain, where this is often explicitly modeled, eg., see Logan and Crump, 2010). Thus, one of the main points of the current manuscript is to highlight the problematic transition from evidence and models that deal with lower level auditory and motoric feedback, to the “personal” level that includes meaning and agency. The type of self-prediction we envisage would be part of a wider self-comprehension skill which uses inferences from many different sources, among them auditory feedback. This position is very similar to the inferential theories that have been proposed in the broader agency literature (such as the bayesian cue-integration account of Moore and Fletcher, 2012). When Wegner and Wheatley (1999) discuss how we determine whether we are the author of our own actions, they place great emphasis on prior thought and expectations in the inferential matrix. Some of the most dramatic examples of malleability of authorship comes from experiments where it is suggested to participants that they have had prior thoughts about outcomes that they actually did not have (e.g., see Wegner and Sparrow, 2004; see also Johansson et al., 2005, 2013; Hall et al., 2012, 2013, for evidence on inferential processing regarding one’s own prior motives).

## Future research directions

In Lind et al. (in press) we used a new type of technique for auditory feedback manipulation, RSE, where semantic aspects of auditory feedback are manipulated in real time. We provided the first empirical demonstration indicating that auditory feedback helps specify, to ourselves, the meaning of what we say. It is our hope that RSE will be adopted as a new tool for psycholinguistic research to approach the difficult questions of conceptualization and self-monitoring, and lead to new avenues in the study of how feedforward and feedback mechanisms interact during speech. To this end, we will make public the detailed specifications of the hardware and software setup of our platform, and the RSE software system will eventually be made freely available upon request (Breidegard et al., in preparation). Below we provide a few suggestions for future work.

One possible future improvement of RSE would be to explore semantic exchanges using existing digital signal-processing techniques which are used to manipulate e.g., formant frequencies or fundamental frequency (e.g., Houde and Jordan, 1998; Jones and Munhall, 2000), instead of the present “record- and insert” technique that we used. Using a digital signal-processing technique one could possibly ensure that word duration, intensity, voice quality and other acoustic parameters are more precisely matched, thereby avoiding any detections of the manipulations that stem from non-linguistic factors. However, the scope of a continuous transformation in real time would be severely limited, and it would be difficult to find meaningful and interesting manipulations (particularly if social contextual factors are to be considered, see below).

One limiting factor of RSE is that it requires that we know in advance what the participants are about to say, otherwise it would be impossible to precision fit a replacement utterance. Thus, we needed a structured task like the Stroop test for the Lind et al. (in press) study. In this context it would not have been informative to ask the participants what they *intended* to say, because the intentions are specified in the task instructions. Both the traditional and the self-comprehension model would make the same predictions about what the participants (would say they) intended to say on any given trial. Unless the participants actively try to foil the experiment, it makes no sense to intend to give the incorrect response.

As discussed above it is not necessary to explicitly ask about intentions to measure their (purported) role in verbal self-monitoring, but in future studies it would nevertheless be highly interesting to try to create a RSE experiment that allowed for the necessary predictability, yet did not instruct the participants what to say (with a bottomless participant pool this could be accomplished just by reasonable guessing and a much higher failure rate than in Lind et al., in press). As the self-comprehension hypothesis places great emphasis on the social nature of speech, this introduces a whole new category of evidence that could both support or work against different word exchanges. During such a situation, social feedback from the experimenters and possible confederates could be controlled with relation to the manipulated feedback. For example, social feedback could be made to align with the manipulated feedback to see if the inserted utterance is more likely to be accepted as self-produced. Or discrepancies can be created between auditory and social feedback to see if the inserted utterance is less likely to be accepted, or if perhaps auditory feedback in such cases can override social feedback. For example, imagine if technical advances had allowed us to make real-time exchanges of spontaneous speech at a dinner party. To test the self-comprehension model we would then be required to incorporate the social reactions and responses of the other guests in the manipulation. Thus, if the host asked our participants if they would fancy another slice of dessert, and we exchanged a “yes” for a “no” (or *vice versa*), whether this insertion would be accepted or not, might to a large degree depend on the social reaction it gets. If the reaction is supportive of the manipulation (the host and other guest loudly approves, saying of course one should have a second helping of this exquisite dessert), then this ought to indicate to the participants that the insertion was a plausible and successful utterance, and that they meant it all along. If, on the other hand, the reactions suggest that the response was somehow inappropriate (if the other guests grumble about not having received their first serving yet), then the participants ought to use this information to distance themselves from the manipulation, and explain they actually meant to decline another serving.

In the example above the hypothetical responses to be manipulated (“yes” and “no”) are phonologically rather more dissimilar than the words “grå” (gray) and “grön” (green), which we used in Lind et al. (in press). If we independently could vary both phonological and semantic similarity in RSE we could explore their relative roles in influencing detection rate. Most likely, it would not be possible to exchange the word “lejon” (lion) for the word “noshörning” (rhinoceros). But it seems safe to assume that “häst” (horse) could be exchanged for “hingst” (stallion), or “fred” (peace) for “frid” (calm/peace), or, slightly more dissimilar “plan” (flat) to “platt” (flat), “rak” (straight) to “rät” (straight) or “kyss” (kiss) to “puss” (kiss). Conversely, what would happen with words that are phonologically similar, but semantically extremely distinct, like “bil” (car) and “pil” (arrow) or “bok” (book) and “bord” (table)? Again, the self-comprehension model contends that whether these words would be detected primarily depends on whether they are contextually appropriate or not. For example, if a manipulation would be coupled with the type of social feedback discussed in the hypothetical dinner example above, we would predict that (congruent) social feedback could counteract or override both semantic and phonological dissimilarity.

Importantly, the RSE method can also be used for studies that do not aim at covertly manipulating speakers’ auditory feedback. For example, an often quoted argument for the existence of an internal monitoring loop is the so-called “v-horizontal”-argument (see e.g., Levelt, 1989). It concerns the fact that erroneous utterances sometimes have very short error-to-cutoff times (<350 ms; Blackmer and Mitton, 1991; Hartsuiker and Kolk, 2001). These errors, it is argued, could not have been detected by the external, auditory loop: given the time needed to comprehend the utterance, realize it was an error and initiate the interruption, the external loop is simply too slow. Instead, it has been postulated that they are detected via an internal loop.

In a recent study (Lind et al., in press) we have approached the issue of the internal monitoring loop by simulating the interruption of “erroneous” utterances during a reading aloud task. Participants are told that, as they perform the task, their auditory feedback will on some random trials be manipulated so that they will say one word, but hear themselves saying another word. When this happens, they are instructed to stop speaking as quickly as they possibly can. This way we separate auditory feedback from all other forms of feedback the speaker receives, including the proposed internal loop. Preliminary results show how slightly more than half of all interruptions are made within 350 ms, and how some, albeit very few, interruptions are made within 100 ms. Since interruptions are made only on manipulated trials, it is unlikely that these interruptions were anticipatory responses. These results present a challenge to the idea that an internal loop must be postulated in order to account for error detections with very brief error-to-cutoff times.

It is an open empirical question to what extent the self-comprehension model extends to other modalities of language production, but there is evidence to suggest that feedback can play a role during writing which is similar to the one played by auditory feedback during speech suggested by our results. For example, Logan and Crump (2010) showed how writers will, in real-time, as they are typing, take credit when the experimenters covertly correct their errors, and also take the blame for errors covertly inserted by the experimenters when in fact they had not made an error. It therefore seems likely that an experiment similar to Lind et al. (in press) could be implemented in a written task, in for example a chat-conversation online.

## Conflict of interest statement

The authors declare that the research was conducted in the absence of any commercial or financial relationships that could be construed as a potential conflict of interest.
